# Supplementary material for: Viruses hijack FPN1 to disrupt iron withholding and suppress host defense
Source: Nat Commun. 2025 Jul 1;16:5912. doi: 10.1038/s41467-025-60031-w (PMC12216596; doi:10.1038/s41467-025-60031-w)
Supplement: Supplementary file 1 — Supplementary Information [file 41467_2025_60031_MOESM1_ESM.pdf]

Viruses hijack FPN1 to disrupt iron withholding and suppress host defense

Li Tong[1,4], Jie Wang[1,4], Yunjin Ma[1], Chunying Wang[1], Yue Fu[1], Qi Li[1], Chengjiang Gao[2], Hui Song[1], Ying Qin[1], Chunyuan Zhao[1,3,*] and Wei Zhao[1,*]

[1]Department of Pathogenic Biology, Key Laboratory of Infection and Immunity of Shandong Province, and Key Laboratory for Experimental Teratology of the Chinese Ministry of Education, School of Basic Medical Science, Cheeloo College of Medicine, Shandong University, Jinan, Shandong, China

[2]Department of Immunology, School of Basic Medical Science, Cheeloo College of Medicine, Shandong University, Jinan, Shandong, China

[3]Department of Cell Biology, School of Basic Medical Science, Cheeloo College of Medicine, Shandong University, Jinan, Shandong, China

[4]These authors contributed equally

[*] Correspondence: cyzhao@sdu.edu.cn (C.Z.); wzhao@sdu.edu.cn (W.Z.)

Supplementary materials

Table S1. List of oligonucleotides, related to Methods and Figure 1-7.

Name	Prime	Sequence
m*Actb*	Forward	5′-TGACCTTTCAAATGCAGTAGATTCA-3′
	Reverse	5′-TGTTACCAACTGGGACGAC-3′
m*Ifnb*	Forward	5′-ATGAGTGGTGGTTGCAGGC-3′
	Reverse	5′-CTGGGTCATCTTTTCACGGT-3′
m*Ifna4*	Forward	5′-GACTTGTCTGCTACTTGGAATGC-3′
	Reverse	5′-TTGGTTGAGGAAGAGAGGGCT-3′
VSV	Forward	5′-ACGGCGTACTTCCAGATGG-3′
	Reverse	5′-CTCGGTTCAAGATCCAGGT-3′
HSV-1	Forward	5′-ACGACAGTGGCATAGGTTGG-3′
	Reverse	5′-CCGACATCACAAGGGACCTC-3′
HSV-1UL30	Forward	5′-CATCACCGACCCGGAGAGGGAC-3′
	Reverse	5′-GGGCCAGGCGCTTGTTGGTGTA-3′
m*Isg56*	Forward	5′-TGCTGAGATGGACTGTGAGGAA-3′
	Reverse	5′-TCTTGGCGATAGGCTACGACTG-3
m*Isg15*	Forward	5′-AGAAGCAGATTGCCCAGAAG-3′
	Reverse	5′-TGCGTCAGAAAGACCTCATAGA-3′
m*Rantes*	Forward	5′-ATGAACCCAAGTGCTGCCGT-3′
	Reverse	5′-TTAAGGAGCCCTTTTAGACCTTTT-3′
m*Mx1*	Forward	5′-ATGGATTCTGTGAATAATCTGTGCA-3′
	Reverse	5′-CTATGTCTCCAAACTGGGAAGGG-3′
h*IFNB*	Forward	5′-CAACAAGTGTCTCCTCCAAAT-3′
	Reverse	5′-TCTCCTCAGGGATGTCAAAG-3′
h*ACTB*	Forward	5′-GGAAATCGTGCGTGACATTAA-3′
	Reverse	5′-AGGAAGGAAGGCTGGAAGAG-3′
m*Slc40a1*	Forward	5′-TTGCAGGAGTCATTGCTGCTA-3′
	Reverse	5′-TGGAGTTGCACACGATTGAT-3′
mut-*Dtx3l*	Forward	5′-GGCATCGCTGTCATCGCTATGGACACCATTAGTAACAAA-3

	Reverse	′
		5′-CTTTTCCTTCTTGTCCAGTTCTGAG′
mut-*TBK1*	Forward	5′-CAGTGGATGTTCAAATGAGAGAA-3′
	Reverse	5′-TTCTCTCATTTGAACATCCACTGCACGAAGGAAGCTTATG
		T
		TATTAAATACT-3′

siRNA- Sequence

Name	siRNA	Sequence
mSlc40a1		5′-GCAGGAGUCAUUGCUGCUA-3′
mDtx3l	①	5′-GAGGGAACCAUGUCUUACU-3′
	②	5′-CGGGUAGACUAGAGUUCAU-3′
	③	5′-GUCGAUUCAACACGCUUUA-3′
Ctrl		5′-UUCUCCGAACGUGUCACGU-3′

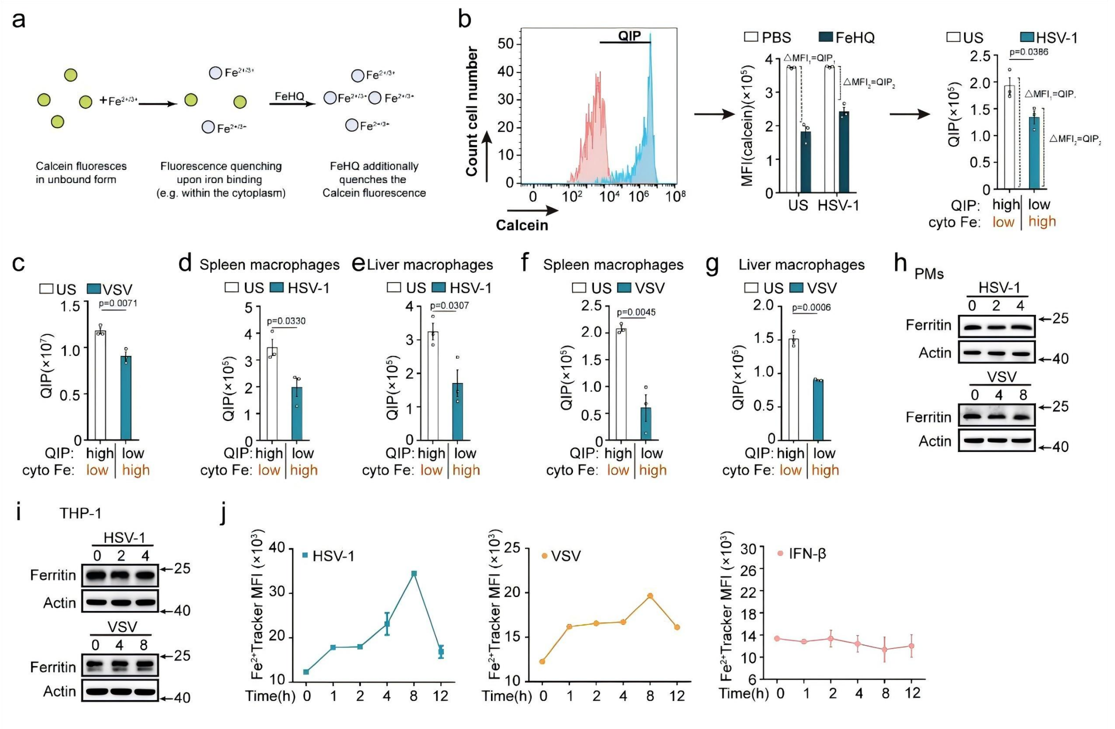

Supplementary figure 1. Viral infection increases cellular ferrous iron.

(a) Schematic representation of the quenchable iron pool (QIP) analysis. Calcein-AM fluoresced in unbound form and fluorescence quenching after iron binding. Furthermore, fluorescence could be totally quenched when additional exogenous FeHQ added to cells. Thereby, the differences in cellular Calcein fluorescence before and after FeHQ addition represent the quenchable iron pool (QIP) of a given cell.

(b) Scheme for QIP calculation. PMs were infected with HSV-1, and the median fluorescence intensity (MFI) of Calcein was determined in naïve (PBS) and FeHQ- challenged PMs, whereby the QIP was calculated as the MFI difference.

(c) QIP analysis of intracellular free iron in PMs infected with VSV.

(d–g) QIP analysis of splenic macrophages (gate in CD11b^{+} Ly6C^{low} F4/80^{+} cells) (d, f) or hepatic macrophages (gate in F4/80^{+} cells) (e, g) infected with HSV-1 (2×10^{7} PFU/mouse) for 4 h (d, e) or VSV (1×10^{9} PFU/mouse) for 8 h (f, g) in C57BL/6 mice. Each symbol represents

one mouse. The flow cytometry strategy is in Fig. S9a, b.

(h, i) Western blot analysis of Ferritin in PMs (h) and in human THP-1 cells (i) infected with HSV-1 or VSV.

(j) Flow cytometry analysis of Fe^{2+} Tracker in PMs infected with HSV-1, VSV, or stimulated with IFN-β (10 ng/mL). The flow cytometry strategy is in Fig. S9c.

Data are expressed as the mean ± SEM in b–g, j. Statistical analyses were performed using two-tailed unpaired Student's t-test. Results were obtained from three independent experiments. US, unstimulated. CKO, *Slac40a1*^{CKO}. Unless otherwise specified, cells infection with HSV-1 (MOI:10) for 4 h, VSV (MOI:1) for 8 h. Lysates marker sizes in kDa are indicated on the western blot right. Source data are provided as a Source Data file.

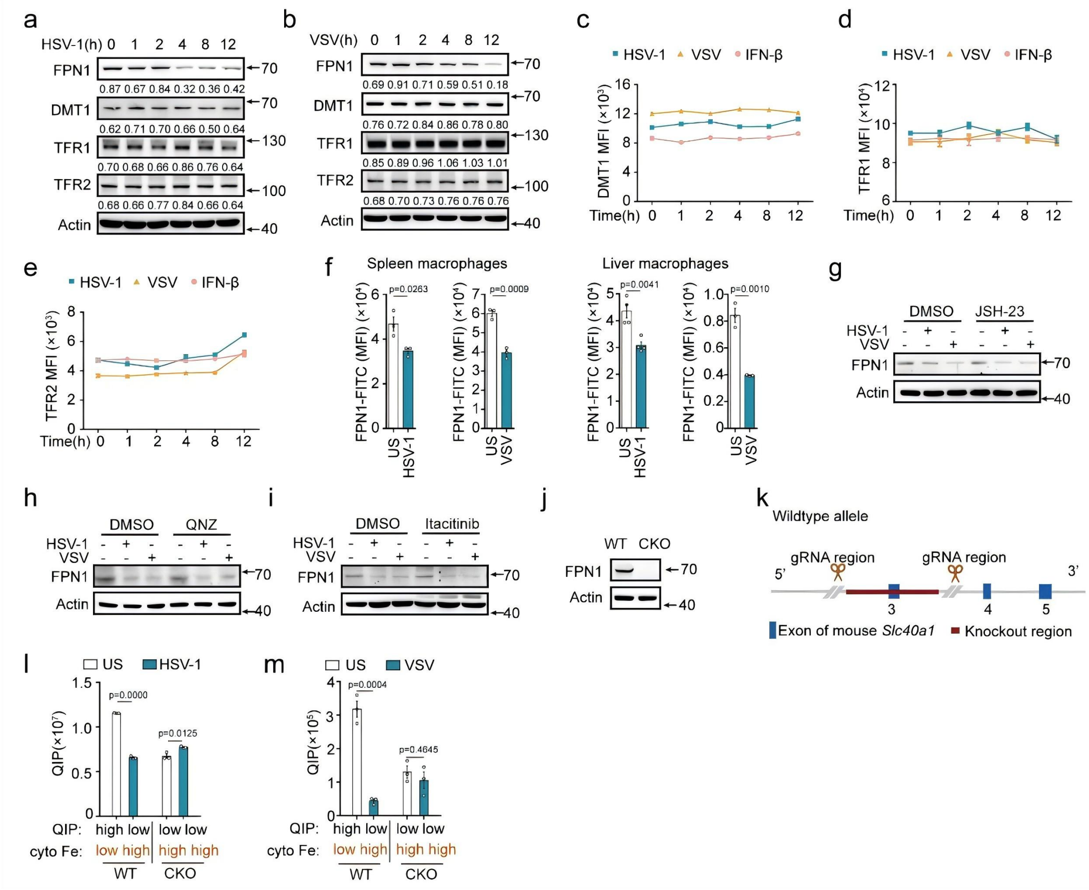

Supplementary figure 2. Viral infection inhibits FPN1 expression.

(a, b) Western blot analysis of DMT1, TFR1, TFR2, and FPN1 in human THP-1 cells infected with HSV-1 (a) or VSV (b).

(c–e) Flow cytometry analysis of the median fluorescence intensity (MFI) of DMT1, TFR1, TFR2 on cell surface in mouse PMs infected with HSV-1 or VSV. Each MFI of FITC or APC was calculated using FlowJo_V10. n=3. The flow cytometry strategy is in Fig. S9f.

(f) Flow cytometry analysis of the MFI of FPN1-FITC in splenic macrophages (gate in $CD11b^{+}$ $Ly6C^{low}$ $F4/80^{+}$ cells) or hepatic macrophages (gate in $F4/80^{+}$ cells) infected with HSV-1 (2×10^{7} PFU/mouse) for 4 h or VSV (1×10^{9} PFU/mouse) for 8 h in C57BL/6 mice. Each symbol represents one mouse. The flow cytometry strategy is in Fig. S9a, b, f.

(g–i) Western blot analysis of FPN1 in mouse PMs treated with JSH-23 (10 μM) (g), QNZ

(10 μM) (h) or itacitinib (10 μM) (i) for 12 h, followed by stimulation with HSV-1 or VSV.

(j) Western blot analysis of FPN1 in wild-type (WT) or *Slac40a1*^{CKO} mouse PMs.

(k) Structure diagram of the knockout region in *Slc40a1*^{CKO} mice, provided by Cyagen Biosciences Inc. (Guangzhou, China).

(l, m) QIP analysis of intracellular ferrous iron in WT or *Slc40a1*^{CKO} mouse PMs infected with HSV-1 or VSV.

Data are expressed as the mean ± SEM in f, l and m. Statistical analyses were performed using two-tailed unpaired Student's t-test. Results were obtained from three independent experiments. US, unstimulated. CKO, *Slac40a1*^{CKO}. Unless otherwise specified, cells infection with HSV-1 (MOI:10) for 4 h, VSV (MOI:1) for 8 h. Lysates marker sizes in kDa are indicated on the western blot right. Source data are provided as a Source Data file.

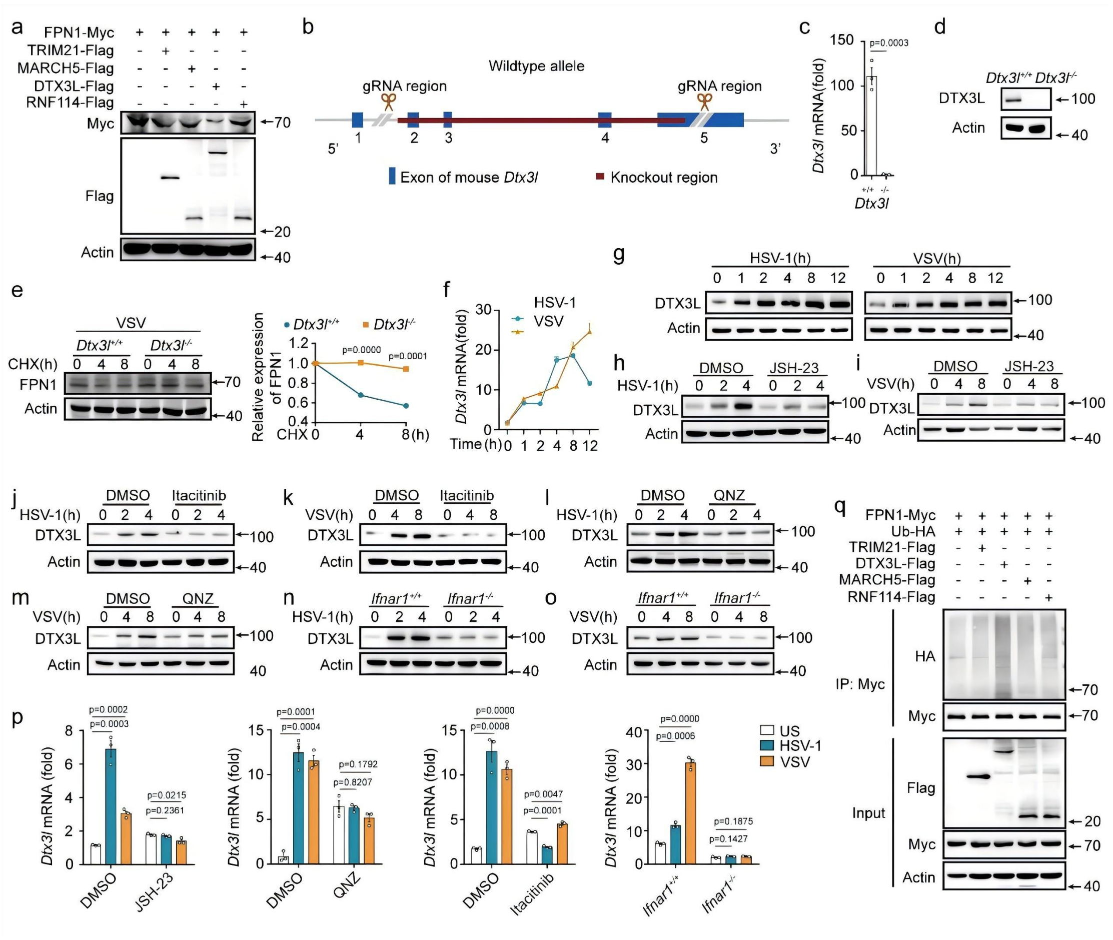

Supplementary figure 3. DTX3L promotes ubiquitous degradation of FPN1.

(a)Western blot analysis of lysates from HEK293T cells transfected with Myc-FPN1 and Flag-TRIM21, Flag-MARCH5, Flag-DTX3L, or Flag-RNF114 plasmids.

(b) Structure diagram of the knockout region in *Dtx3l*$^{-/-}$ mice, provided by Cyagen Biosciences Inc. (Guangzhou, China).

(c, d) RT-PCR (c) and western blot analysis (d) of DTX3L expression in *Dtx3l*$^{+/+}$or *Dtx3l*$^{-/-}$ mouse PMs.

(e) Western blot analysis of FPN1 expression in mouse PMs from *Dtx3l*$^{+/+}$ or *Dtx3l*$^{-/-}$ mice infected with VSV, together with cycloheximide (CHX, 10 μM) for indicated time periods (left). FPN1 expression level was quantified by measuring the band intensities using the "ImageJ" software (right).

(f, g) RT-PCR (f) and western blot (g) analysis of DTX3L expression in HSV-1 or VSV infected mouse PMs.

(h–o) Western blot analysis of DTX3L in mouse PMs treated with dimethyl sulfoxide (DMSO), JSH-23 (10 μM) (h, i), itacitinib (10 μM) (j, k), or QNZ (10 μM) (l, m) for 12 h, or in *Ifnar1*$^{+/+}$ or *Ifnar1*$^{-/-}$ mouse PMs, followed by infection with HSV-1(h, j, l, n) or VSV (i, k, m, o).

(p) RT-PCR analysis of *Dtx3l* mRNA expression in mouse PMs pretreated with DMSO, itacitinib (10 μM), JSH-23 (10 μM), QNZ (10 μM) for 12 h, or in *Ifnar1*$^{+/+}$ or *Ifnar1*$^{-/-}$ mouse PMs, followed by infection with HSV-1 or VSV.

(q) Immunoprecipitation (IP) analysis of the ubiquitination of FPN1 in HEK293T cells transfected with plasmids encoding HA-Ub, Flag-TRIM21, Flag-MARCH5, Flag-DTX3L, and Flag-RNF114, as well as Myc-FPN1, with MG132 (10 μM) treatment for 4 h.

Data are expressed as the mean ± SEM in c, e, f, and p. Statistical analyses were performed using two-tailed unpaired Student's t-test. Results were obtained from three independent experiments. Unless otherwise specified, cells infection with HSV-1 (MOI:10) for 4 h, VSV (MOI:1) for 8 h. Lysates marker sizes in kDa are indicated on the western blot right. Source data are provided as a Source Data file.

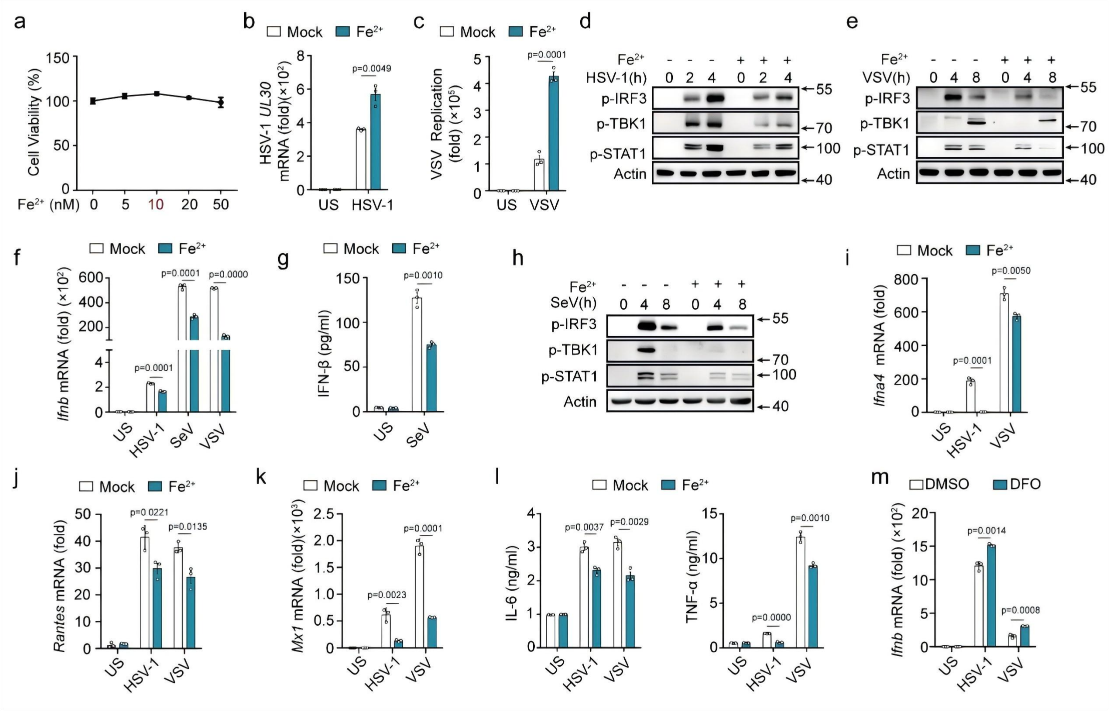

Supplementary figure 4. Ferrous iron attenuates type I IFN responses and autophagy to facilitate viral replication.

(a) Cell Counting Kit-8 (CCK-8) analysis of cell viability of mouse PMs pretreated with increasing amounts of Fe^{2+} (1, 5, 10, 20, and 50 nM) for 12 h.

(b, c) RT-PCR analysis of the *HSV-1 UL30* mRNA or the replication of VSV in PMs, pretreated with LAL water (Mock) or Fe^{2+} (10 nM) for 1 h, followed by infection with HSV-1 (MOI: 10) for 8 h (b) or VSV (MOI: 1) for 12 h (c).

(d–e) Western blot analysis indicated proteins in mouse PMs pretreated with LAL water (Mock) or Fe^{2+} (10 nM) for 1 h, followed by infection with HSV-1 or VSV.

(f) RT-PCR analysis of *Ifnb* mRNA in PMs, pretreated with LAL water (Mock) or Fe^{2+} (10 nM) for 1 h, followed by infection with HSV-1, SeV or VSV.

(g, h) ELISA assay of IFN-β secretion (g) and western blot analysis of indicated proteins (h) in mouse PMs, pretreated with LAL water (Mock) or Fe^{2+} (10 nM) for 1 h, followed by

infection with SeV.

(i–k) RT-PCR analysis of *Ifna4* (i), *Rantes* (j), and *Mx1* (k) mRNA in mouse PMs, pretreated with LAL water (Mock) or Fe^{2+} (10nM) for 1 h, followed by infection with HSV-1 (MOI: 10) for 8 h or VSV (MOI: 1) for 12 h.

(l) ELISA assay of IL-6 secretion and TNF-α secretion in mouse PMs, pretreated with LAL water (Mock) or Fe^{2+} (10 nM) for 1 h, followed by HSV-1 or VSV.

(m) RT-PCR analysis of *Ifnb* mRNA in PMs, pretreated with DMSO or DFO (10 μM) for 4 h, followed by HSV-1 or VSV.

Data are expressed as the mean ± SEM in b, c, f, j, and i–m. Statistical analyses were performed using two-tailed unpaired Student's t-test. Results were obtained from three independent experiments. US, unstimulated. Unless otherwise specified, cells infection with HSV-1 (MOI:10) for 4 h, VSV (MOI:1) for 8 h, SeV (MOI:1) for 8 h. Lysates marker sizes in kDa are indicated on the western blot right. Source data are provided as a Source Data file.

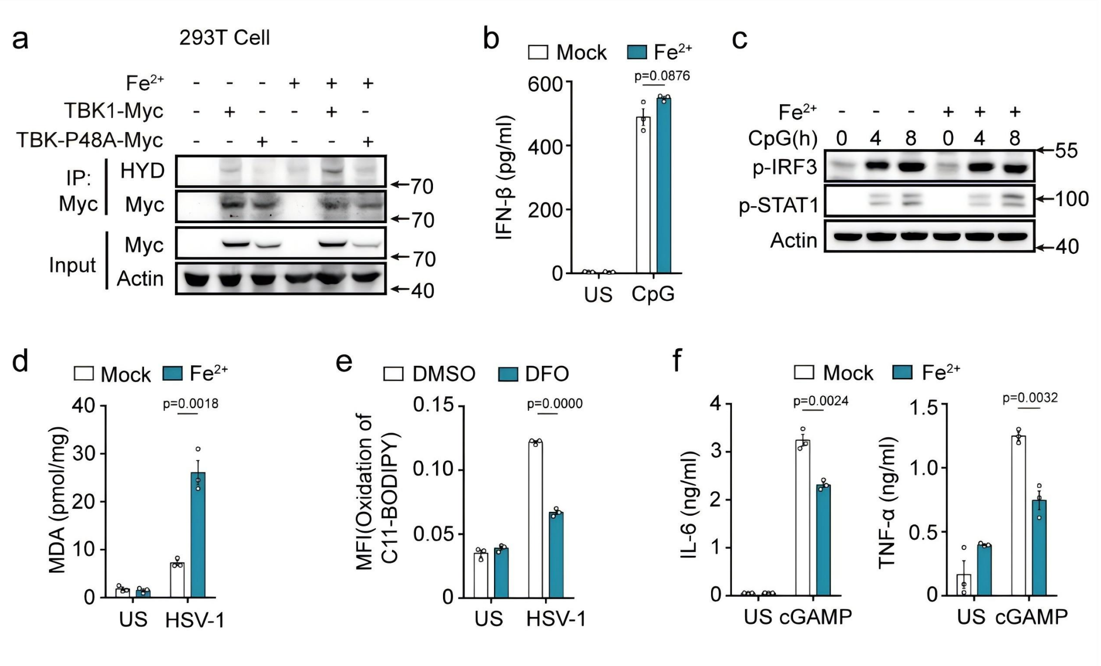

Supplementary figure 5. Ferrous iron enhances TBK1 prolyl hydroxylation and STING carbonylation.

(a) Western blot assays of TBK1 prolyl hydroxylation (HYD) in HEK293T cells transfected with TBK1-Myc and TBK1-P48A-Myc and pretreated with LAL water (Mock) or Fe^{2+} (10 nM) for 1 h, followed by immunoprecipitation with Myc antibody.

(b, c) ELISA analysis of IFN-β secretion (b) or western blot assays of p-TBK1, p-IRF3, and p-STAT1 (c) in PMs from WT or *Slc40a1*^{CKO} mice and then transfected with CpG (1μg/mL) for 8 h.

(d) Malondialdehyde (MDA) production in mouse PMs pretreated with LAL water (Mock) or Fe^{2+} (10 nM) for 1 h, followed by infected with HSV-1.

(e) Flow cytometry analysis of lipid peroxidation in mouse PMs pretreated with DMSO or DFO (10 μM) for 4 h, and then infected with HSV-1. Lipid peroxidation is calculated as the MFI (FITC/PE) of lipid peroxidation sensor C11-BODIPY.

(f) ELISA assays of IL-6 and TNF-α secretion in mouse PMs pretreated with LAL water

(Mock) or Fe^{2+} (10 nM) for 1 h, and then transfected with cGAMP (5μg/mL) for 4 h.

Data are expressed as the mean ± SEM in b and d–f. Statistical analyses were performed using two-tailed unpaired Student's t-test. Results were obtained from three independent experiments. US, unstimulated. Unless otherwise specified, cells infection with HSV-1 (MOI:10) for 4 h. Lysates marker sizes in kDa are indicated on the western blot right. Source data are provided as a Source Data file.

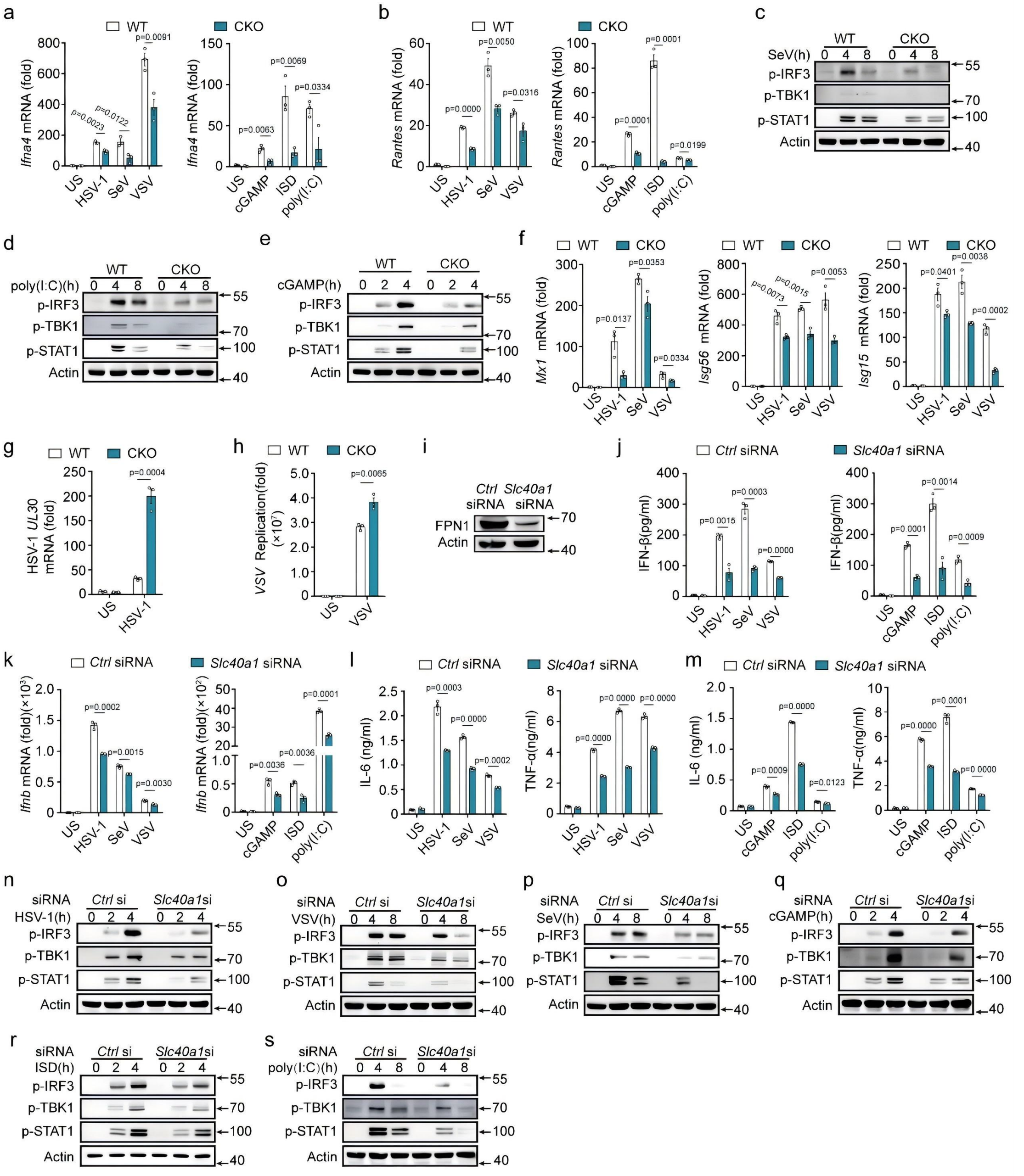

Supplementary figure 6. FPN1 enhances type I IFN responses and autophagy.

(a–e) RT-PCR analysis of *Ifna4* mRNA expression (a) or *Rantes* mRNA expression (b) and western blot assays of p-TBK1, p-IRF3, and p-STAT1 (c-e) in PMs from WT or *Slc40a1*^{CKO} mice, followed by infected with HSV-1, SeV, VSV, transfected with cGAMP (5μg/mL) for 4 h, ISD (10μg/mL) for 4 h, or poly(I:C) (10μg/mL) for 8 h.

(f–h) RT-PCR analysis of *Isg15, Isg56* and *Mx1* mRNA expression (f), the *HSV-1 UL30*

mRNA (g) or the replication of VSV (h) in PMs from WT or *Slc40a1*^{CKO} mice, followed by infection with HSV-1 (MOI: 10) for 8 h, SeV (MOI:1) for 12 h or VSV (MOI: 1) for 12 h.

(i) Western blot analysis of FPN1 in Ctrl siRNA or *Slc40a1* siRNA-transfected mouse PMs.

(j, k) ELISA assays (j) and RT-PCR assays (k) of IFN-β expression in Ctrl siRNA or *Slc40a1* siRNA-transfected mouse PMs, followed by infected with HSV-1, SeV, VSV, cGAMP (5μg/mL) transfected for 4 h, ISD (10μg/mL) transfected for 4 h or poly(I:C) (10μg/mL) transfected for 8 h.

(l, m) ELISA assays of IL-6, and TNF-α secretion in Ctrl siRNA or *Slc40a1* siRNA-transfected mouse PMs, followed by infection with HSV-1, SeV and VSV (l), or transfected with cGAMP (5μg/mL) for 4 h, ISD (10μg/mL) for 4 h and poly(I:C) (10μg/mL) for 8 h (m).

(n–s) Western blot analysis of p-IRF3, p-TBK1, and p-STAT1 in Ctrl siRNA or *Slc40a1* siRNA-transfected mouse PMs, followed by infection with HSV-1 (n), VSV (o), SeV (p), transfected with cGAMP (5μg/mL) (q), ISD (10μg/mL) (r) or poly(I:C) (10μg/mL) (s).

Data are expressed as the mean ± SEM in a, b, f–h, and j–m. Statistical analyses were performed using two-tailed unpaired Student's t-test. Results were obtained from three independent experiments. US, unstimulated. CKO, *Slc40a1*^{CKO}. Unless otherwise specified, cells infection with HSV-1 (MOI:10) for 4 h, VSV (MOI:1) for 8 h, SeV (MOI:1) for 8 h. Lysates marker sizes in kDa are indicated on the western blot right. Source data are provided as a Source Data file.

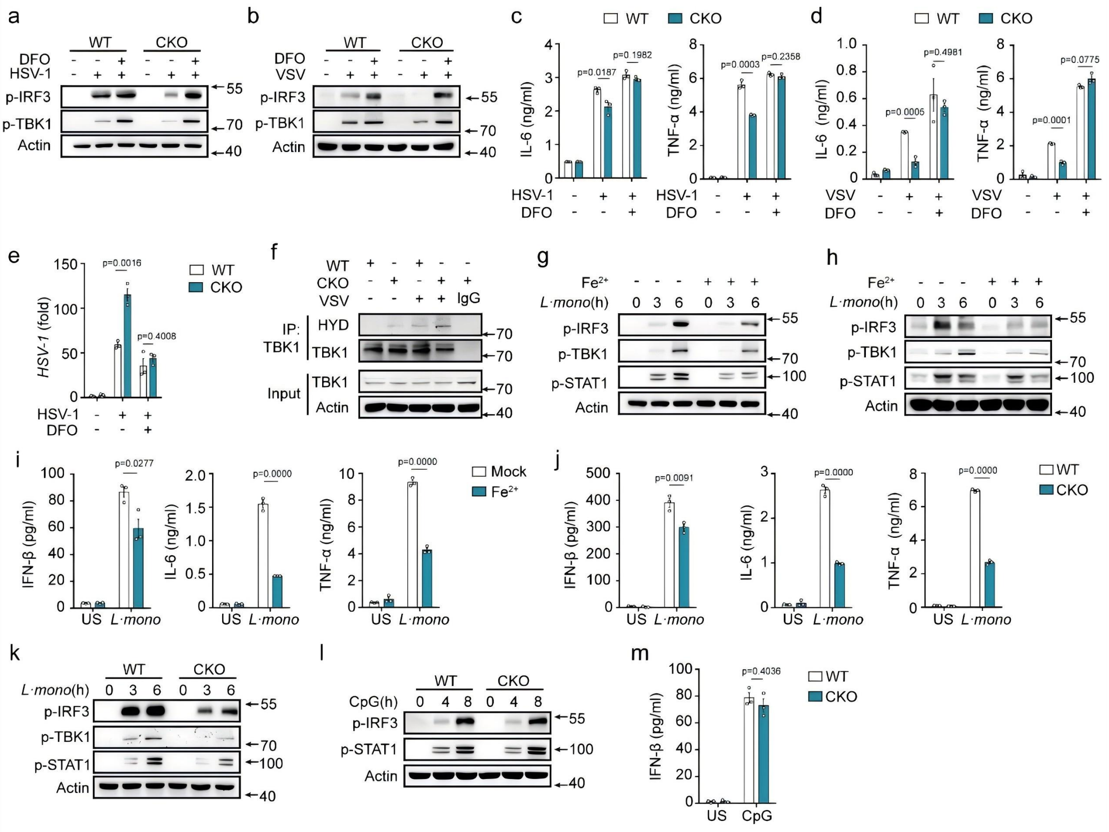

Supplementary figure 7. FPN1 inhibits viral replication *in vitro* and *in vivo*

(a–d) Western blot assays of p-TBK1, p-IRF3, and p-STAT1 (a, b) or ELISA analysis of IL-6 and TNF-α secretion (c, d) in PMs from WT or *Slc40a1*^{CKO} mice pretreated with DMSO or DFO (10 μM) for 4 h, followed by infection with HSV-1 (a, c) or VSV (b, d).

(e) RT-PCR analysis of the replication of HSV-1 in PMs from WT or *Slc40a1*^{CKO} mice pretreated with DMSO or DFO (10 μM) for 4 h, followed by infection with HSV-1.

(f) Western blot assays of TBK1 prolyl hydroxylation (HYD) in PMs from WT or *Slc40a1*^{CKO}mice treated with VSV infection, followed by immunoprecipitation with TBK1 antibody.

(g, h) Western blot analysis indicated the presence of proteins in PMs (g) or THP-1 cells (h) pretreated with LAL water (Mock) or Fe^{2+} (10 nM) for 1 h, followed by infection with *L. mono*.

(i) ELISA assays IFN-β, IL-6 and TNF-α secretion in mouse PMs, pretreated with LAL water (Mock) or Fe^{2+} (10 nM) for 1 h, followed by infection with *L. mono.* for 6 h.

(j, k) ELISA analysis of IFN-β, IL-6, and TNF-α secretion, and western blot assays of p-TBK1, p-IRF3, and p-STAT1 (k) in PMs from WT or *Slc40a1*^{CKO} mice, followed by infection with *L. mono.* for 6 h.

(l, m) Western blot assays of p-TBK1, p-IRF3, and p-STAT1 (l) and ELISA analysis of IFN-β secretion (m) in PMs from WT or *Slc40a1*^{CKO} mice, followed by transfection with CpG (1μg/mL) for 8 h.

Data are expressed as the mean ± SEM in c–e, i–j, and m. Statistical analyses were performed using two-tailed unpaired Student's t-test. Results were obtained from three independent experiments. US, unstimulated. *L. mono*., *Listeria monocytogenes.* CKO, *Slc40a1*^{CKO}. Unless otherwise specified, cells infection with HSV-1 (MOI:10) for 4 h, VSV (MOI:1) for 8 h. Lysates marker sizes in kDa are indicated on the western blot right. Source data are provided as a Source Data file.

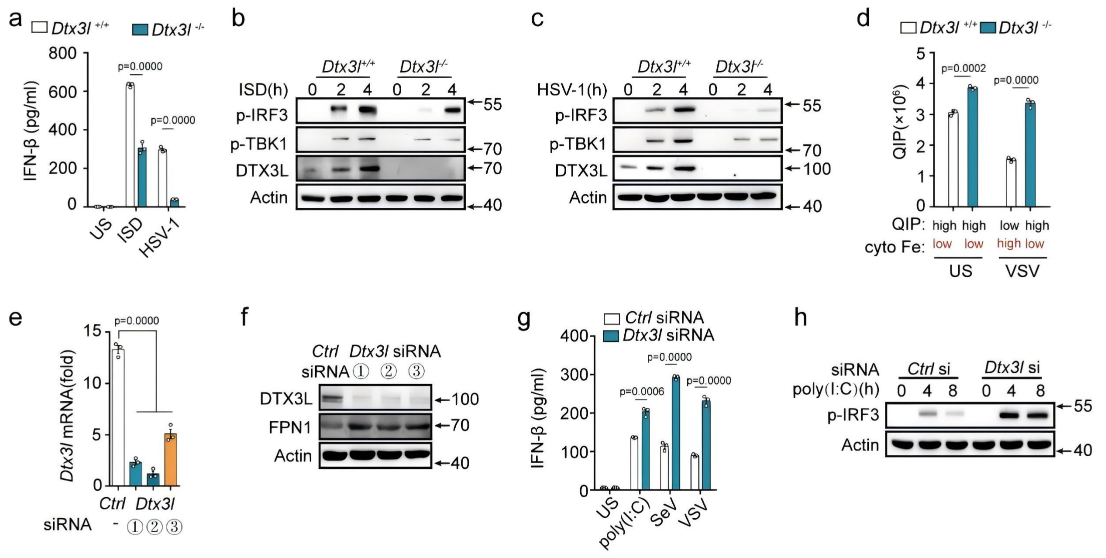

Supplementary figure 8. DTX3L attenuates innate responses against RNA viruses.

(a) ELISA analysis of IFN-β secretion in PMs from *Dtx3l*$^{+/+}$or *Dtx3l*$^{-/-}$ mice, followed by transfection with ISD (10 μg/mL) for 4 h or infection with HSV-1.

(b, c) Western blot assays of p-TBK1, p-IRF3, and p-STAT1 in PMs from *Dtx3l*$^{+/+}$or *Dtx3l*$^{-/-}$ mice, followed by transfection with ISD (10 μg/mL) (b) or HSV-1 (c).

(d) QIP analysis of intracellular free iron in *Dtx3l*$^{+/+}$or *Dtx3l*$^{-/-}$ mouse PMs infected with VSV.

(e) RT-PCR analysis of *Dtx3l* expression in Ctrl siRNA or *Dtx3l* siRNA-transfected mouse PMs.

(f) Western blot analysis of FPN1 and DTX3L in Ctrl siRNA or *Dtx3l* siRNA-transfected mouse PMs.

(g) ELISA assays IFN-β secretion in Ctrl siRNA or *Dtx3l* siRNA-transfected mouse PMs, followed by stimulation with poly(I:C) (10 μg/mL) for 8 h, infection with VSV or SeV.

(h) Western blot analysis of p-IRF3 in Ctrl siRNA or *Dtx3l* siRNA-transfected mouse PMs, followed by transfection with poly(I:C) (10 μg/mL).

Data are expressed as the mean ± SEM in a, d, e, and g. Statistical analyses were performed using two-tailed unpaired Student's t-test. Results were obtained from three independent experiments. US, unstimulated. Unless otherwise specified, cells infection with HSV-1 (MOI:10) for 4 h, SeV (MOI:1) for 8 h, VSV (MOI:1) for 8 h. Lysates marker sizes in kDa are indicated on the western blot right. Source data are provided as a Source Data file.

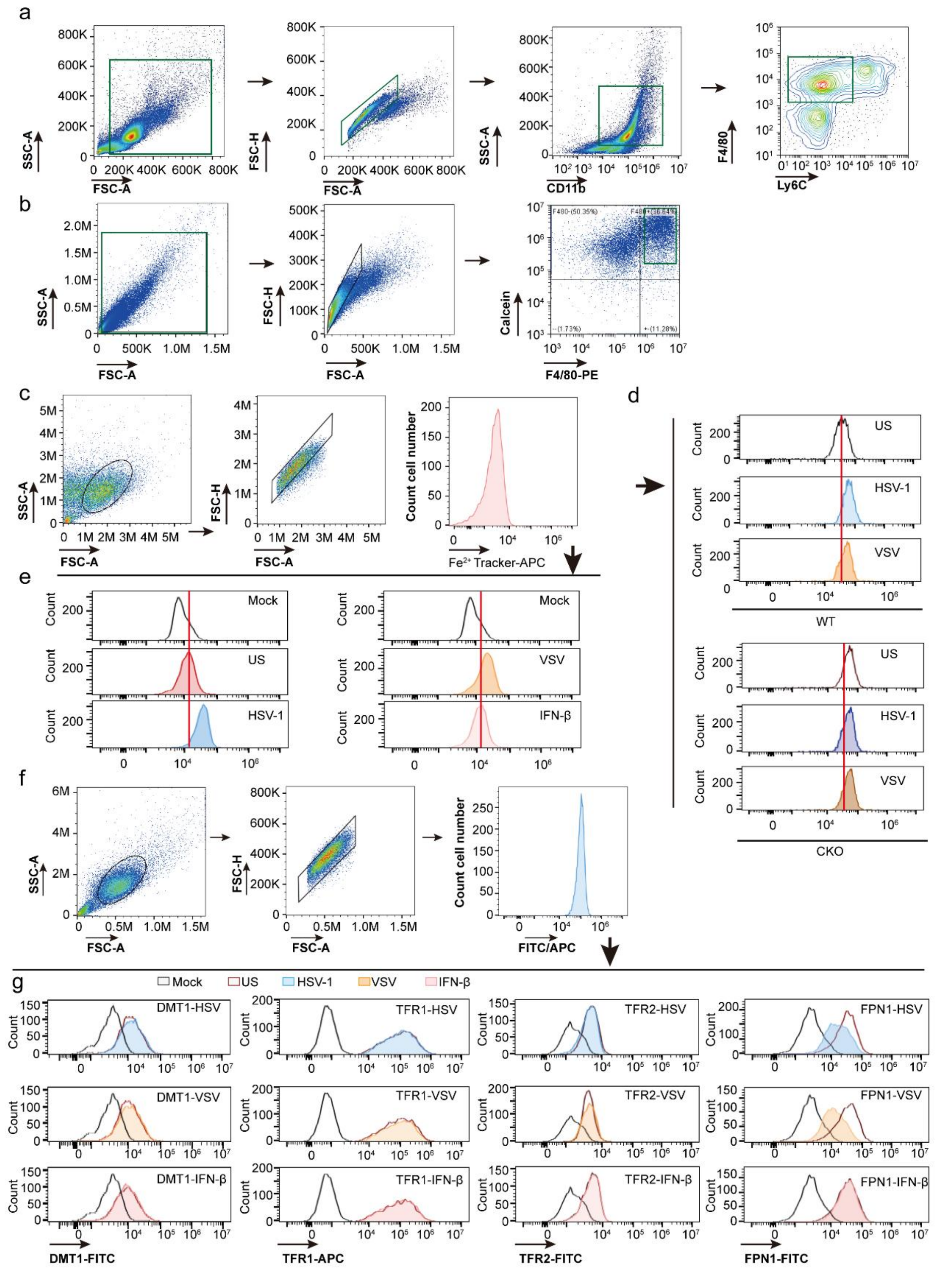

Supplementary figure 9. flow cytometry analyses gating strategies

(a, b) Gating strategy for identifying myeloid cell subsets in the spleen (a, gate in $F4/80^{+}CD11b^{+}$ $Ly6C^{low}$ cells) or liver (b, gate in $F4/80^{+}$cells) during virus infection in Fig. S1d–g, S2f.

(c) Flow cytometry strategy for identifying macrophages was used in all panels from Fig. 1l, Fig. S1j.

(d) Representative flow cytometry results for determination of Fe^{2+} Tracker in Fig.1l.

(e) Representative flow cytometry results for determination of Fe^{2+} Tracker in Fig.S1j.

(f) Flow cytometry strategy for identifying macrophages was used in all panels from Fig. 1h, Fig.S2c-e.

(g) Representative flow cytometry results for determination of FPN1/DMT1/TFR2/TFR1 expression in Fig. 1h, Fig.S2c-e.

Mock, blank control. US, unstimulated.
